# EHMTI-0253, syncopal migraine: factors that influence the positive response to tilt table testing

**DOI:** 10.1186/1129-2377-15-S1-E31

**Published:** 2014-09-18

**Authors:** G Corcea, S Odobescu, O Grosu, C Chicu-Hadarca, D Concescu, I Moldovanu, L Rotaru

**Affiliations:** 1 Neurology, Institute of Neurology and Neurosurgery, Chisinau, Moldova

## Background

Syncopal migraine is a condition when patient has migraine and syncope simultaneously (Curfman, 2012). Both conditions are known to present with autonomic imbalance that may determine a positive response to tilt table testing.

## Aim

was to evaluate the factors that could influence the tilt table response in syncopal migraine.

## Method

The study sample consisted of 65 patients with syncopal migraine. All the patients underwent detailed clinical examination and tilt table testing using Westminster protocol. According to the response on tilt test, the patients were divided in two groups and compared: Gr. I (N=32) with positive response and Gr. II (N=33) with negative response. All the data collected were analyzed using SPSS software.

## Results

In the syncopal migraine sample, 50.7% of the patients had positive response to the test (developing presyncopal or syncopal symptomatology during the test). The first group presented increased frequency of headache-days per month (9.18 vs. 3.13, p<0.001), more severe headaches (8.1 vs. 6.7, VAS, p<0.05), more expressed anxiety and depression, according to Spilberger test (36.5±1.7 vs. 29.26±1.7, p<0.05) and depression Beck score (8.03±0.09 vs. 5.01±0.6, p<0.05), respectively. The Nijmegen score was very high in the group I (31.56±1.4 vs. 15.29±1.0, p<0.001) indicating the presence of a respiratory dysfunctional syndrome.

## Conclusion

Frequent and severe headaches, increased anxiety and depression, the presence of dysfunctional respiratory syndrome could be the factors that predispose syncopal migraine patients to positive response on tilt table testing, which is an orthostatic stress and indicate autonomic imbalance and increased cardiovascular reactivity.

No conflict of interest.

